# Ethical Challenges and Lessons Learned During the Clinical Development of a Group A Meningococcal Conjugate Vaccine

**DOI:** 10.1093/cid/civ598

**Published:** 2015-11-09

**Authors:** Lionel Martellet, Samba O. Sow, Aldiouma Diallo, Abraham Hodgson, Beate Kampmann, Siddhivinayak Hirve, Milagritos Tapia, Fadima Cheick Haidara, Assane Ndiaye, Bou Diarra, Patrick Odum Ansah, Adebayo Akinsola, Olubukola T. Idoko, Richard A. Adegbola, Ashish Bavdekar, Sanjay Juvekar, Simonetta Viviani, Godwin C. Enwere, Elisa Marchetti, Julie Chaumont, Marie-Francoise Makadi, Flore Pallardy, Prasad S. Kulkarni, Marie-Pierre Preziosi, F. Marc LaForce

**Affiliations:** 1 Meningitis Vaccine Project, PATH, Ferney-Voltaire, France; 2 Centre pour le Développement des Vaccins, Bamako, Mali; 3 Institut pour la Recherche et le Développement, Niakhar, Senegal; 4 Navrongo Health Research Centre, Ghana Health Service, Navrongo, Ghana; 5 Vaccines and Immunity Theme, Medical Research Council Unit, Basse, The Gambia; 6 Shirdi Sai Baba Hospital, Vadu/King Edward Memorial Hospital Research Centre, Rasta Peth, Pune, India; 7 Department of Pediatrics, Center for Vaccine Development, University of Maryland School of Medicine, Baltimore; 8 GlaxoSmithKline Vaccines, Wavre, Belgium; 9 Serum Institute of India, Ltd, Pune; 10 Meningitis Vaccine Project, Department of Immunization, Vaccines and Biologicals, World Health Organization, Geneva, Switzerland

**Keywords:** informed consent, ethics, subject protection, ethics committees, developing countries

## Abstract

***Background.*** The group A meningococcal vaccine (PsA-TT) clinical development plan included clinical trials in India and in the West African region between 2005 and 2013. During this period, the Meningitis Vaccine Project (MVP) accumulated substantial experience in the ethical conduct of research to the highest standards.

***Methods.*** Because of the public–private nature of the sponsorship of these trials and the extensive international collaboration with partners from a diverse setting of countries, the ethical review process was complex and required strategic, timely, and attentive communication to ensure the smooth review and approval for the clinical studies. Investigators and their site teams fostered strong community relationships prior to, during, and after the studies to ensure the involvement and the ownership of the research by the participating populations. As the clinical work proceeded, investigators and sponsors responded to specific questions of informed consent, pregnancy testing, healthcare, disease prevention, and posttrial access.

***Results.*** Key factors that led to success included (1) constant dialogue between partners to explore and answer all ethical questions; (2) alertness and preparedness for emerging ethical questions during the research and in the context of evolving international ethics standards; and (3) care to assure that approaches were acceptable in the diverse community contexts.

***Conclusions.*** Many of the ethical issues encountered during the PsA-TT clinical development are familiar to groups conducting field trials in different cultural settings. The successful approaches used by the MVP clinical team offer useful examples of how these problems were resolved.

***Clinical Trials Registration.*** ISRCTN17662153 (PsA-TT-001); ISRTCN78147026 (PsA-TT-002); ISRCTN87739946 (PsA-TT-003); ISRCTN46335400 (PsA-TT-003a); ISRCTN82484612 (PsA-TT-004); CTRI/2009/091/000368 (PsA-TT-005); PACTR ATMR2010030001913177 (PsA-TT-006); PACTR201110000328305 (PsA-TT-007).

Between 2005 and 2013, the Meningitis Vaccine Project (MVP) and partners conducted 9 clinical studies to evaluate a new group A meningococcal conjugate vaccine (PsA-TT), manufactured at the Serum Institute of India, Ltd, in Pune, India. The clinical studies enrolled and followed >11 000 participants at 8 clinical trial sites in The Gambia, Ghana, India, Mali, and Senegal. All MVP clinical studies were conducted following the Declaration of Helsinki, the International Conference of Harmonisation–Good Clinical Practice guidelines (ICH-GCP), and in accordance with other international, national, and local guidelines as applicable [1–4]. Because of the scope and location of this clinical program, MVP and its partners have accumulated valuable experience in resolving ethical questions that arose during the preparation and conduct of the studies. This article highlights the ethical review process, community permission, informed consent, and other ethical issues that were encountered during the clinical program.

## ETHICAL REVIEWS

### Responsible Ethics Committees for MVP Clinical Studies

Because MVP was a partnership between the World Health Organization (WHO) and PATH, all MVP clinical trial proposals were reviewed by the WHO Research Ethics Review Committee, Switzerland (WHO-ERC), and by the PATH Research Ethics Committee (PATH-REC) or the Western Institutional Review Board (WIRB), an independent ethics committee (EC) based in the United States. In addition, for all clinical sites, approvals were obtained from national or institutional ECs and the national regulatory authorities (NRAs) of respective countries.

**Figure 1.**
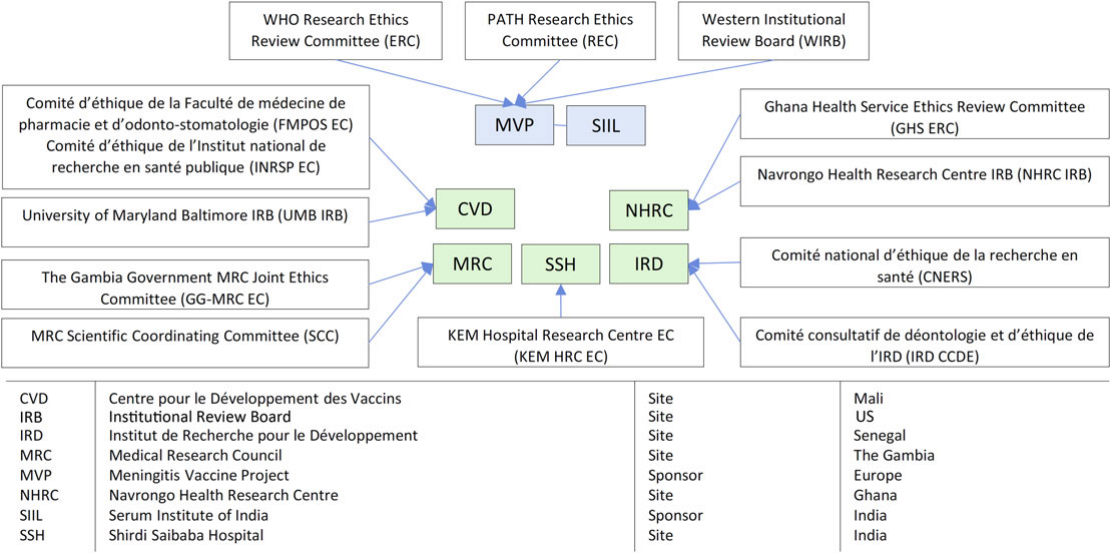
Sponsor and site ethics committees in the Phase 2 and Phase 3 clinical development of the group A meningococcal conjugate vaccine (PsA-TT). Abbreviation: WHO, World Health Organization.

Figure 1 provides an overview of the ethical landscape for the PsA-TT clinical trials. Every submission and communication with the ECs was handled in close collaboration with the sponsor study representative and the principal investigator. It was important to speak with one voice to the ECs to ensure that any recommended changes or issues raised by an EC would also be communicated to other ECs. The sponsors and the investigators were key to making this happen through joint correspondence and teleconferences (designated groups are listed in Figure 1). The end result was that we routinely provided feedback not only to local ECs but, in a timely fashion, to WHO-ERC, PATH-REC, and WIRB.

### MVP Ethical and Regulatory Submission Strategy

Because of the number of ethics committees involved, a proper sequence of submissions and reviews was essential; the plan was developed in advance and carefully reassessed along the way to ensure that targets for study initiation were met. Presubmission dialogue with ECs was critical to ensure that the timing and the format of the research proposals were acceptable.

The preferred strategy was to submit to the sponsor ECs first whenever possible (PATH-REC or WIRB), then integrate their comments into a new proposal, which would then be submitted to the investigator ECs and to the WHO-ERC simultaneously. For multisite studies, the proposals were usually submitted in parallel to ECs of different investigator sites. Sponsor ECs would, however, not grant final approval until approvals from all investigator ECs had been granted. After all EC approvals were obtained, NRA submissions were made.

PATH institutional review board reviewers acted as a primary filter before releasing the proposals to the WHO-ERC and country site ECs. Tracked amendment forms enabled ECs of sponsor and different investigator sites to have access to changes and their rationale, following the preceding reviews, and supported the sustained dialogue between all parties. This stepwise approach and continuous interchange led to a more efficient review, with fewer revisions to study documents and fewer resubmissions.

### Postsubmission Dialogue

After each review, the sponsors (PATH and SIIL), together with investigators, maintained a dialogue with the ECs, especially when there were comments or questions. It was essential that each comment and its implications were clearly understood by all parties so that responses clearly addressed all questions. Follow-up communication by email and telephone was very valuable, in particular, when a new collaboration with an EC was begun.

## COMMUNITY PERMISSION

The success of any clinical study is largely dependent on community and participant support. The importance of community involvement in international clinical trials has become increasingly recognized [5–8].

Beyond obtaining the formal approvals from regulatory, ethical, and scientific authorities, it was essential to solicit the active support of the communities in India and West Africa where the studies were taking place. In these traditional societies, particularly in rural areas, community voices included the community elders, religious leaders, elected councils, women's associations, traditional doctors, and local criers.

Initial community meetings prepared by the study investigators in collaboration with partners established a format for dialogue between the research team and the population, which was maintained throughout the study. The research staff, investigators, and fieldworkers, who often belonged to these communities, played a critical role in keeping the communities engaged [5].

Once studies were completed, the results were shared with the community through community feedback/dissemination meetings. At these meetings, investigators presented the results of the study. These meetings gave an opportunity for the study team to address questions and provide recommendations based on findings and to thank the community and the participants for their contributions. The feedback meetings also helped assure community ownership and the possibility of future collaborations [5].

## INFORMED CONSENT

As per the Declaration of Helsinki, ICH-GCP, and the regulatory guidelines, informed consent is critical to ensure the protection and the rights of potential research participants who come into contact with the research team. In MVP studies, informed consent was taken from all the participants ≥18 years of age. For children and adolescents (1–17 years), parents/legal guardians gave the consent. In addition, written assent was taken from the participants for older children aged 12–17 years, following the local EC recommendations. When dealing with illiterate participants/parents/legal guardians, an impartial literate witness, independent from the investigator team, attested the consent process.

### Translation to the Local Languages

Original informed consent forms were drafted and reviewed in English; once finalized, appropriate translations were prepared at specific sites. For Mali and Senegal, French translations were developed. In India, translations in the Hindi, Marathi, Urdu, and Telugu languages were developed. These translations were back-translated to English and validated following a standard operating procedure (SOP) before being submitted to ECs. This validation process ensured that the translations were done correctly.

In the early stages of the project, the same process was followed to obtain formal translations and back-translations of the informed consent forms in the local dialects of West Africa (particularly in Mali, Senegal, The Gambia, and Ghana). Consent forms were prepared in the following local languages: Bambara, Fula, Wollof, Mandinka, Sarahule, Nankam, Kassem, and Serer.

However, the local dialect translations posed 2 important difficulties:

- The local dialects are verbal languages and not commonly used in writing. Few people study their written form. The participants and the study teams felt more comfortable in using the forms in the official language of their country (French in Mali and in Senegal; English in The Gambia and in Ghana) rather than those in local dialects.
- The back-translation posed a significant challenge because the local dialects have different roots from Western languages. Translators had to rely on the use of metaphors and cultural references to translate Western vocabulary into the local dialects. Such constructions had the potential to lose their meaning in the back-translation, which posed greater challenges.

Due to the constraints surrounding writing of local dialects, study sites used the following alternative approach (reviewed and approved by ECs) to ensure the consent in the local languages.

### Informed Consent as an Interactive Process

West African oral traditions rely heavily on the value of spoken communication. “You have my word” is perceived as a more powerful statement than a written signature. Participants expected some form of interaction and discussion to be able to ascertain implications of their participation in the study.

Therefore, site teams used several methods and materials to make the process easily understandable in the local dialects. Teams developed tape recordings, transcripts of recordings in oral language, and specific trainings for study staff to convey in a consistent manner consent information in the local languages. Training sessions were documented; recordings and transcripts were certified, reviewed, and approved by the local EC, although these were not the literal translations of English forms.

Fieldworkers were fully trained on how to communicate the elements of consent. An impartial literate witness ensured that an oral communication matched information present in the consent form.

After verbal discussion in the field, the research team shared copies of the informed consent forms (in English or French) with potential participants and their families. In some sites, during a first visit to the clinic, potential participants were given the opportunity to consult with their family and literate relatives before making a decision.

During later studies, to ensure a good understanding of the information provided prior to consenting, some of the study sites began using assessment of understanding tools to evaluate the comprehension of the study by the potential participant prior to consent. This allowed the research team to revisit the study information with the participant in case some aspects had not been well understood.

Investigators and their research teams ensured a sound approach for informed consent process using the above-mentioned process. Such elements have emerged in the literature as proven approaches for the process of informed consent [6, 7, 9, 10]. The success of the informed consent process still required the investigators' continued transparency, confidentiality, and respect of the participant in the implementation of these tools, while ensuring a thorough documentation of each informed consent.

## PRACTICAL ISSUES

The following are some special situations that arose during the trials.

### Pregnancy Testing at Study Entry

From 2009 to 2011, at the Center for Vaccine Development, Bamako, Mali, MVP conducted a large phase 3 study in the healthy population aged 1–29 years. The study required a specific exclusion criterion to prevent the enrollment of pregnant women. To comply, the study procedures included a negative pregnancy test before vaccination for all women of childbearing potential (ie, postmenarcheal and/or married women).

Initially, the protocol was reviewed by the WHO-ERC, which requested precision on how to identify postmenarcheal women, how assent would be done for minors, and what would ensue in case of positive pregnancy test results in minors. The WHO-ERC comments raised some challenging ethical questions to the research team: How to request a pregnancy test from minors and unmarried women? How to ensure the confidentiality of the test result in this group? How to manage the social consequences of the positive test result for the participant and her family? How to ensure that the process of pregnancy testing/counseling is well accepted by the community?

These questions led to detailed exchanges between the local EC, the WHO-ERC, the investigators, and the MVP responsible medical officer. The following approach satisfied the WHO-ERC and the site EC and was culturally acceptable within the Malian community context: An SOP for pregnancy testing was developed by the study site that defined the informed consent process and enrollment for women aged ≥18 years, for women aged 13–17 years, and for girls aged 10–12 years. In each of these age groups, a different approach was taken to ensure the confidentiality and respect of the potential participant. A counseling office was established to meet privately all women and young girls entering the study. Following the SOP, trained midwives consulted privately with the minor girl/woman about the need for a pregnancy test to enter the study and whether they would be willing to take the test. The decision to accept or refuse the test was kept entirely confidential. The strategy proved to be successful. The discretion offered by the midwife counseling was well accepted by the community and helped assure participation of minors in the study. Over time, the midwife consultation became a popular demand among the community.

This was an important lesson on how best to address an ethical issue raised by a defined research protocol. By working openly and collectively with all parties, the issue was successfully resolved.

### Provision of Care

A clinical study involves health assessment of the participants at periodic clinical visits. During the assessments, health problems unrelated to the study may be uncovered. In such cases, the common practice is to provide medical referrals under the assumption that participants will be able to obtain care using their medical insurance coverage or from the public health system, or last, will pay medical costs. The provision of ancillary care in clinical trials has become an important issue in international clinical research because access to healthcare varies considerably from country to country [7, 11–13].

Some of our sites had governmental healthcare plans and others had community pharmacies or charity clinics that provide basic care. However, the care provided at such different sites may not be optimal and could put study participants at risk. Though not a legal requirement, the sponsors considered it a moral obligation to provide care and treatment to the participants even in case of unrelated issues, in compliance with the guidance principles provided in international ethical guidelines [2, 3, 14, 15]. To ensure uniform medical care, for any related or unrelated acute health issue diagnosed during the study, immediate treatment was provided free of charge to every participant as per standard of care in the country. Investigators would work closely with the local healthcare providers to ensure adequate reimbursement of costs for participants, including purchasing family health insurance for trial participants when available in the participating community.

As the number of trials and the number of participants increased over time, the investigators were confronted with a few serious illnesses in study participants. Examples included a subject diagnosed with leukemia; a participant becoming paraplegic after a tree fall; and a diagnosis of human immunodeficiency virus infection. For each of these cases, the sponsor covered the cost of any treatment and rehabilitation for the duration of the study. At the end of the study, the sponsor worked with the investigators to facilitate adequate referral, long-term support, and any follow-up.

The investigators were also confronted during the course of our studies with broader health signals. In Ghana, for example, a significant number of malaria cases were encountered in the study population. Across Ghana, Mali, and The Gambia, the investigators were faced with cases of serious malnutrition, often related to harvest/climatic conditions and/or political instability. This led the sponsor and the concerned site investigators to take action for the subjects and the communities affected. This is described in more detail elsewhere in this supplement [5].

All of the above examples illustrate how research teams and sponsors of clinical studies can be confronted with medical issues that extend beyond the traditional role of a researcher or a sponsor but require attention.

### Posttrial Access

Because our trials involved the administration of a study vaccine (PsA-TT, MenAfriVac), we could not guarantee benefit from any protection against meningitis A after receipt of the investigational vaccine. Therefore, we planned to offer a dose of the licensed polysaccharide vaccine to subjects at the end of the studies.

However, when the immunogenicity and safety profile of the study vaccine became further characterized after the release of the phase 2/3 trial results, 4 weeks after immunization, the study vaccine was found to perform better than the licensed vaccine, triggering the MVP Expert Panel to recommend that the PsA-TT conjugate vaccine be offered to all participants who had not received it during the trial to be protected against meningitis A. This recommendation posed a critical challenge; favorable preliminary results from clinical trials were available, yet this vaccine had not reached a reasonably sized safety database and its regulatory file was not yet ready for licensure submission.

Nevertheless, we decided to proceed with this recommendation by amending all relevant study protocols. At the end of the original study, subjects with low immunogenicity responses were offered the possibility to receive either a dose of the licensed ACWY polysaccharide vaccine or a dose of PsA-TT vaccine and be followed for an additional 3 months to monitor safety parameters. This approach was accepted by the Project Advisory Group and approved by all concerned ECs. There were, however, logistical and timeline implications, and considerable additional resources were needed to proceed with all ethical and regulatory clearances as well as to support adequate field implementation. Nevertheless, our trials were taking place in areas where epidemic meningitis caused by group A was a major health risk, and the risk–benefit ratio had become highly favorable to promote its access to study participants in compliance with the guiding principles of international ethical guidelines [16].

For later trials, the PsA-TT conjugate vaccine was fully licensed for the 1- to 29-year-olds. By the time these later trials drew to a close, we could offer the PsA-TT outside study procedures to all subjects within this age category without any particular safety follow-up. In addition, in the following years, all countries where clinical trials were implemented introduced the vaccine through national campaigns, which provided coverage to all communities who participated in the trials.

This issue of timely access of participating communities to an investigational product is a common concern in international clinical trials [8, 11] and was raised during the MVP development. Our response was probably imperfect and raised additional logistical challenges. However, it does illustrate the need to develop approaches early in study plans for posttrial access that is reasonable to the communities and in line with sponsor resources without compromising safety and scientific objectives.

## CONCLUSIONS

The diverse cultures encompassed in the study sites meant that a regional ethical approach could not necessarily work in the same manner in each site. Hence, alternative approaches were developed to respect the social and cultural context of the community. Respect, listening, and working collectively were all equally important to make things happen. Building from past experiences across cultures, the investigators and the sponsors were sensitive to these aspects from the start. When different parties had differing beliefs about the right way to deal with a situation, discussion and exchanges were essential to understand one another's point of view. With patience and dialogue, a common ground could be built to develop research proposals.

The field of ethics in international clinical trials is a dynamic environment. The experiences that are faced in the field continue to inform levels of regulatory and ethical scrutiny. During 8 years of MVP clinical trials, we sensed an increased demand for real field questions. While collaborating with diverse ethics committees, we benefited from complementary perspectives as well as the experience of navigating through multiple review processes in a continuous, mutually enriching dialogue to the benefit of the quality of research and attention to participating communities. Investigators and sponsors accumulated experiences and challenges that proved to be of interest to ECs, advisory committees, and independent safety monitoring boards. We shared our experiences on the informed consent process, community and participant healthcare, disease prevention, aspects of counseling, and confidentiality within diverse cultural settings, in an effort to enrich the ethical context of the MVP trials.
